# MineSDS: A Unified Framework for Small Object Detection and Drivable Area Segmentation for Open-Pit Mining Scenario

**DOI:** 10.3390/s23135977

**Published:** 2023-06-27

**Authors:** Yong Liu, Cheng Li, Jiade Huang, Ming Gao

**Affiliations:** 1 Zhuzhou CRRC Times Electric Co., Ltd., Zhuzhou 412001, China; 2 State Key Laboratory of Advanced Design and Manufacturing for Vehicle Body, College of Mechanical and Vehicle Engineering, Hunan University, Changsha 410082, China

**Keywords:** autonomous driving, small object detection, drivable area segmentation, open-pit mine

## Abstract

To tackle the challenges posed by dense small objects and fuzzy boundaries on unstructured roads in the mining scenario, we proposed an end-to-end small object detection and drivable area segmentation framework for open-pit mining. We employed a convolutional network backbone as a feature extractor for both two tasks, as multi-task learning yielded promising results in autonomous driving perception. To address small object detection, we introduced a lightweight attention module that allowed our network to focus more on the spatial and channel dimensions of small objects without impeding inference time. We also used a convolutional block attention module in the drivable area segmentation subnetwork, which assigned more weight to road boundaries to improve feature mapping capabilities. Furthermore, to improve our network perception accuracy of both tasks, we used weighted summation when designing the loss function. We validated the effectiveness of our approach by testing it on pre-collected mining data which were called Minescape. Our detection results on the Minescape dataset showed 87.8% mAP index, which was 9.3% higher than state-of-the-art algorithms. Our segmentation results surpassed the comparison algorithm by 1 percent in MIoU index. Our experimental results demonstrated that our approach achieves competitive performance.

## 1. Introduction

Image-based object detection is a critical task of autonomous driving perception [1], providing essential environmental information for decision-making and planning of autonomous vehicles. In mining operation situation, autonomous driving technology for mining trucks can provide valuable security guarantees for workers. However, current environment perception technology primarily aims for generalization in common scenarios and overlooks applicability for special scenarios, such as mines and ports. Designing a perception algorithm based on the particular scene’s characteristics is the foundation for improving perception in the corresponding scene. In a general mining environment, numerous small stones cluster on the surface without road lane markings, making it challenging to identify drivable areas with indistinct boundaries [2]. These issues pose significant challenges to visual perception methods, which can negatively impact mining truck driving safety. Deploying algorithms on autonomous vehicles necessitates balancing limited computational resources with low latency requirements. Compared to sequential methods, multi-task networks with a shared backbone for each task can handle tasks in parallel. Thereby, it is necessary to design an efficient object detection and drivable area segmentation joint framework for these two typical tasks of the mining scene so as to improve the perception capability of the mining truck.

Recent vision-based object detection methods, such as YOLO [3] and CornerNet [4] showed remarkable behavior in structed urban road. In relation to ordinary object detection in structed urban road, small stones are arranged more densely, irregularly, easily occluded and their appearance are similar to the background in the mining situation. Generally, small objects are defined as objects with pixels less than 32 × 32 in pictures [5]. In the past few years, small object detection (SOD) attracted the attention of researchers. Many representative studies emerged such as data-manipulation methods, feature-fusion methods, scale-aware methods and context-modeling methods [6]. In terms of data-manipulation, RRNet [7] invented AdaResampling, an adaptive resampling strategy for copying and pasting small objects in image, to improve detection accuracy. Feature pyramid network (FPN) [8] designed a bottom-up and top-down network, which can construct feature maps of different scales. FA-SSD [9] used additional features in different depth levels of the network as context, supplemented by attention mechanism to focus on the object in the image. The above methods achieved competitive results. However, there is still a large gap between the performance of these methods and that of general object detection.

In addition, drivable area segmentation for the mining environment is also a challenging task which need to be solved. The drivable area, which is entirely distinct from the structured urban road, has indefinite shape features, unstable color features and variable texture features. Recently, deep convolutional neural networks were used to handle the drivable area segmentation [10]. Some algorithms were proposed to deploy the segmentation methods directly on autonomous vehicles. For instance, ref. [11] proposed the dual-resolution networks for real-time semantic segmentation of road-driving images. Ref. [12] regarded the recognition of the drivable area as a row-selection task, which significantly reduced the computational costs. The ability of these algorithms in mining scenarios remains need to be tested. The above algorithms were designed to solve the assignments of SOD and semantic segmentation; thus, there is no general and end-to-end framework for mining environment detection and drivable area segmentation.

To summarize, we proposed a novel framework that tackled the autonomous mining vehicles challenges faced by small object detection and drivable area segmentation (also called mineSDS). Our contributions include: (1) An end-to-end framework capable of concurrently processing object detection and drivable area segmentation tasks. Our framework employs a shared feature extractor to extract features for both tasks, thereby reducing inference time and computational costs. (2) A lightweight attention module designed to enhance the detection accuracy of small objects. By enabling our network to pay more attention to small objects in the spatial and channel dimensions, we achieved improved detection performance. (3) We incorporate a convolutional block attention module to enhance the feature mapping ability of convolutional operations in the segmentation decoder, improving our network’s sensitivity to fuzzy road boundaries. Our framework’s performance was evaluated on the Minescape dataset, and our results demonstrated comparable performance in both tasks.

The remainder of this paper is organized as follows: related works are summarized in Section 2; the details of our proposed algorithm are described in Section 3. Additionally, the experimental analysis is demonstrated on Section 4. At last, the contributions of this paper are summarized on Section 5.

## 2. Related Works

In recent years, significant progress was made in small object detection and drivable area segmentation. This section review related work about mine scene perception, small object detection and drivable area segmentation.

### 2.1. Open-Pit Mining Perception

In the field of mine scene detection, Ren et al. [13] proposed a multi-scale feature fusion method for obstacle detection of roads in open pit mining areas, normalized the features, compressed the features and extracted multi-scale features. Song et al. [14] used multi-scale and attention fusion modules to capture richer context features on their own data sets and achieved good results in the field of mine target detection. For mining scene segmentation, Wei et al. [15] extracted multi-scale features by a multi-branch feature extraction network. Xiao et al. [16] designed RATT-UNet network to extract open-pit mine roads information. Liu et al. [17] used point cloud data to track mine road.

### 2.2. Small Object Detection

Small object detection made great progress in recent years. The researchers mainly made improvements to solve problems as follows: (1) the feature information of small object is extremely lacking, (2) small gravel objects are more densely arranged, more irregular, and more easily blocked. First of all, many studies made improvements in the data-manipulation methods. The most direct way is to increase the number of small objects in the data set. Ref. [18] first proposed the strategy of randomly copying small objects in the data set, simply and directly increasing the number of small objects. On this foundation, Chen et al. proposed the [7] network to determine the background of small objects before copying and pasting small object samples. In addition, Duan, Wei et al. [19,20] proposed a variety of ways to use mosaic to solve the small object detection problem, and achieved certain results. The mosaic data augmentation method was proposed in YOLOv4 [21] paper. The main idea is to randomly cut four pictures and then splice them into one picture as training data. The advantage of this is that it enriches the background of image, and the four images are spliced together to improve the batch size in a disguised way, four images will also be calculated during batch normalization.

For small objects, the most important thing is to complete the feature extraction at the corresponding scale. Dollár [8] used an image pyramid with a sliding window scheme to extract features. Yang et al. [22] selected a feature layer of appropriate size for the pooling operation of small object features. Cai et al. proposed the MS-CNN [23] method to select the best reception region for small object objects in different layer scales. In the solution of feature fusion, Lin et al. [8] first proposed a feature pyramid network (FPN), the purpose of which is to aggregate high resolution semantic features with higher level features in low resolution. This simple and effective design became an important part of the feature extractor. Zand et al. built DarkNet RI [24] on the basis of DarkNet-53 and skip-connection to generate high-level semantic feature maps of different scales.

Using attention mechanism is also a common way for small object detection. Its main principle is to use the weight relationship to obtain the feature information that should pay more attention. SE Attention [25] module is the comparative attention, which is characterized by adding attention mechanism in the dimension of special channel. ECA-Net [26] module effectively captures cross channel interaction by using one-dimensional convolution. CBAM (convolutional block attention module) [27] starts from the two scopes of channel and spatial, introduces two analysis dimensions of spatial attention and channel attention and realizes the sequential attention structure from channel to space.

### 2.3. Drivable Area Segmentation

Semantic segmentation involves pixel-level classification of dense image prediction. The problem of early semantic segmentation is that it cannot effectively extract high-level semantic features, and the effect is poor on complex images. The fixed receptive field cannot match the scale change of the object, and simple up-sampling will lead to the loss of detailed information. Long et al. first proposed a network (FCN) [28] that can process features on images of arbitrary resolution. U-Net [29] proposed a novel u-shape network structure, in which the down-sampling image features are fused with the up-sampling information and the pixel level features are segmented to avoid the loss of pixel level semantic features. Chen et al. proposed DeepLab [30,31], which first used dilation convolution to increase receptive field, effectively avoiding the problem of feature disappearance in down-sampling.

Drivable area segmentation derives from semantic segmentation. Drivable area segmentation task is a special task of semantic segmentation. Travelable area segmentation is mainly used for the perception of autonomous vehicle. Due to the particularity of the task and the characteristics of large range and clear edge features of the drivable area compared with complex segmentation targets, drivable area segmentation requires more accuracy and real-time. In the aspect of drivable area segmentation, the YOLOP [32] panoramic perception network completed three times of up-sampling on the basis of YOLO framework to achieve the segmentation of drivable area. YOLOPv2 [33] uses a new mixed loss function to improve the segmentation and detection effects. HybridNets [34] uses the efficient segmentation head of the weighted bidirectional feature network to perform segmentation, which is an accurate and practical method. Hong et al. proposed DDRNet [11], which uses deep dual-resolution networks are composed of two deep branches between which multiple bilateral fuels are performed. Asgarian et al. [12] consider the process of drivable area recognition as a row-selection task, increasing the speed of performance. Wang et al. added C6 and C7 modules with larger receiving fields on the basis of YOLACT [35] to improve the segmentation of the drivable area.

In order to better detect small obstacles on the road surface, we presented two networks: (1) we added a combination of SimAM and channel attention CAM (channel attention module) module on the last layer of the in the redesigned YOLOv7 head, which achieved better detection results. (2) In terms of segmentation, five up-samples followed by RepbottleneckCSPA and CBAM were used. As for the above two points, we proposed MineSDS, which can simultaneously meet the requirements of obstacle detection in the mining area and segmentation of the exercisable area.

## 3. Methodology

**Figure 1:**
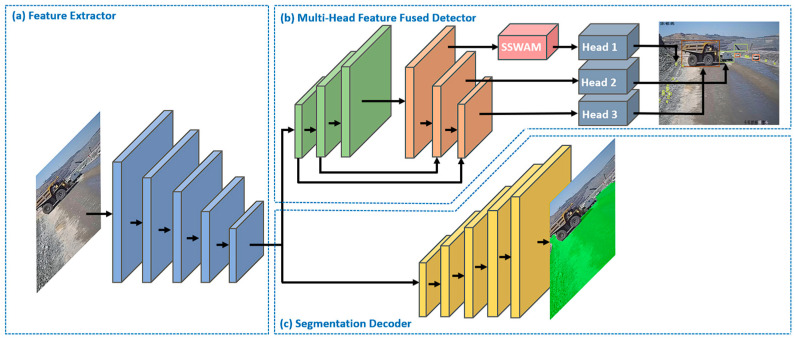
The architecture of MineSDS. (**a**) Feature Extractor. (**b**) Multi-Head Feature Fused Detector. (**c**) Segmentation Decoder.

We put forward a framework to achieve small object detection and semantic segmentation; the details of the proposed method are shown in Figure 1. As the figure shows, our architecture consisted of a (a) feature extractor, (b) segment decoder and (c) multi-head feature fused detector. 

### 3.1. Feature Extractor

**Figure 2:**
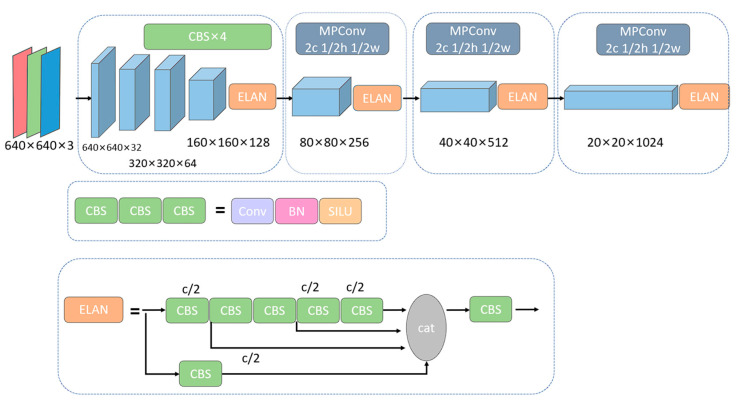
Feature Extractor.

The feature extraction module takes an image as input and outputs semantic features, which can be used as input for subsequent object detection and semantic segmentation. Many representative classification networks were used for feature extractors, such as ResNet [36], DenseNet [37], etc. In the down-sample module, we used both max pooling and convolution with the stride of two. The input image first passed through three convolution modules, and the size of feature map became (H/4, W/4, 128). Then, the feature went into three modules consisting of ELAN module and down-sample module. Finally, the feature was enhanced by an ELAN module. The input image, thus, became a feature map with the size of (H/32, W/32, 1024). The detail of the feature extractor is shown in Figure 2:

### 3.2. Multi-Head Feature Fused Detector

We implemented a multiscale detection scheme in our multi-head detection module, which relied on anchor-based detection. To aggregate features, we used a combination of feature pyramid network (FPN) and path aggregation network (PAN) modules. The feature map was first passed through a top-down feature pyramid, and then through a bottom-up feature pyramid with PAN structure. This allowed us to merge information from different scales and perform object detection at different scales using multiple prediction heads directly on the fused feature map of the PAN. In our approach, each grid on the feature map corresponded to a branching head, which was assigned three anchors of different shapes. Each detection head predicted three tensors encoding the bounding box, category and prediction confidence. By using FPN and PAN, we can fuse features at different scales, which allows the smaller object detection branch to obtain valuable information from earlier feature maps, preventing the loss of information during repetitive down sampling. Furthermore, we proposed a small object attention submodule to enhance detection of smaller objects. This submodule reassigned feature weights to better detect small objects on the branch that detects smaller objects.

In order to address the challenges associated with detecting small objects, we introduced a novel small scale-wise attention module that was capable of reassigning weights for both spatial and channel features. This innovative module comprised two components: SimAM and CAM.

SimAM is a method grounded in neuroscience theory, which employs energy functions to explore the significance of various elements within the feature map. Utilizing SimAM, we were able to derive 3D attention, whereby spatial attention was 2D and channel attention was 1D. The energy function was initially defined in thermodynamics to characterize the stability of a system. It is now widely employed to express the degree of divergence of elements within a system, where smaller energy values correspond to a more stable system. The energy function can be mathematically expressed as:

(1) $$e_{v}\left(w_{v},b_{v},u,x_{i}\right)={\left(u_{v}-\hat{v}\right)}^{2}+\frac{1}{L-1}\overset{L-1}{\underset{i=1}{\sum }}{\left(u_{o}-{\hat{x}}_{i}\right)}^{2}$$

where $\hat{v}=w_{v}v+b_{v}$ represents the feature values of the target elements and $\hat{x_{i}}=w_{v}x_{i}+b_{v}$, which represents the feature values of other elements in the same spatial dimension. i and L=W×H is the index of the feature element and the number of feature element over spatial dimension.

To find the linear separability between the target element $\hat{v}$ and other element ${\hat{x}}_{i}$, it adopts binary labels (i.e., 1 and −1) for $u_{v}$ and $u_{o}$. and also add a regularization term into Equation (1) for simplicity. Then, the final energy function is as follows:

(2) $$e_{v}\left(w_{v},b_{v},u,x_{i}\right)=\frac{1}{L-1}\overset{L-1}{\underset{i=1}{\sum }}{\left(-1-\left(w_{v}x_{i}+b_{v}\right)\right)}^{2}+{\left(1-\left(w_{v}v+b_{v}\right)\right)}^{2}+{\lambda w}_{v}^{2}$$

By minimizing the final energy function, the separability between $\hat{v}$ and ${\hat{x}}_{i}$ can be found. Additionally, the minimal energy can be computed with the following:

(3) $$e_{v}^{\min}=\frac{4\left({\hat{\sigma }}^{2}+\lambda \right)}{{\left(v-\hat{\mu }\right)}^{2}+{2\hat{\sigma }}^{2}+2\lambda }$$

here, $\hat{\mu }=\frac{1}{l}\sum_{i=1}^{L-1}x_{i}$ and ${\hat{\sigma }}^{2}=\frac{1}{L}\sum_{i=1}^{L-1}{\left(x_{i}-\hat{\mu }\right)}^{2}$ the smaller $e_{v}^{\min}$, the more different the element is from the surrounding elements and the higher the corresponding attention weight. We can achieve attentional weight redistribution by:

(4) $$X^{\prime }=\mathrm{sigmoid}\left(\frac{1}{E}\right)⊙X$$

where X*,* $X^{\prime }\in R^{C\times H\times W}$.

With this method, the spatial information of the small object was well attended to by the network.

It was observed that while the attention weights generated by SimAM were 3D, the majority of the attention was assigned to the spatial dimension. To address the reassignment of channel weights to features, which is a common practice in attention methods, we implemented the CAM prior to SimAM. The CAM comprised two parallel branches that performed max-pooling and avg-pooling, respectively. The channel attention weight was then obtained through MLP and normalized using the sigmoid function, before being used to weight each channel in the original input feature map one by one to recalibrate the channel attention to the original feature. The overall channel attention process can be summarized as follows:

(5) $$A_{c}\left(F_{\mathrm{in}}\right)=\sigma (\mathrm{MLP}\left(\mathrm{MaxPool}\left(F_{\mathrm{in}}\right)\right)+\mathrm{MLP}\left(\mathrm{AvgPool}\left(F_{\mathrm{in}}\right)\right))$$

(6) $$F_{\mathrm{out}}=A_{c}\left(F_{in}\right)⊗F_{in}$$

$F_{\mathrm{in}}$ and $F_{\mathrm{out}}$ are the input feature map and output feature map, $F_{\mathrm{in}}$, $F_{\mathrm{out}}\in R^{C\times H\times W}$. MLP is multilayer perceptron. 

### 3.3. Segmentation Decoder

**Figure 3:**
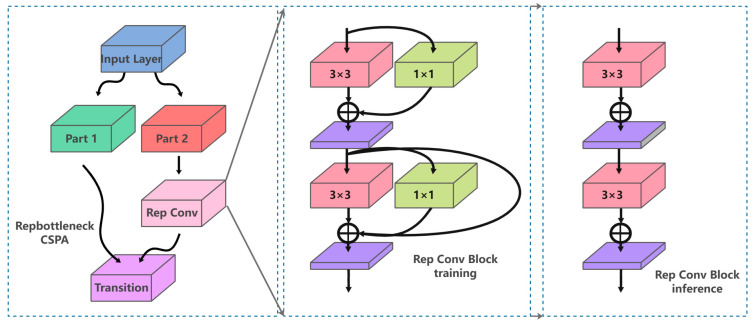
RepBotterneckCSP.

The segmentation decoder is responsible for assigning a label (drivable area or background) to each pixel in the input image. The input feature map with a size of “(H/32, W/32, 1024)” was fed into the segmentation decoding module. After performing five up-sampling module operations, the output feature map was restored to the original image size, representing the probabilities of each pixel being part of the background or drivable area. Our up-sampling module included an up-sampling operation and a RepBottleneckCSP module, which employed the CSPNet architecture to connect rep convolution groups. The CSP architecture enabled richer gradient combinations while reducing computational complexity, and the RepConv structure improved fitting ability by using a multichannel structure during training and a single-channel structure during inference, making the RepBotterneckCSP module a popular choice for many mainstream networks. The structure of RepBotterneckCSP is shown in Figure 3.

**Figure 4:**
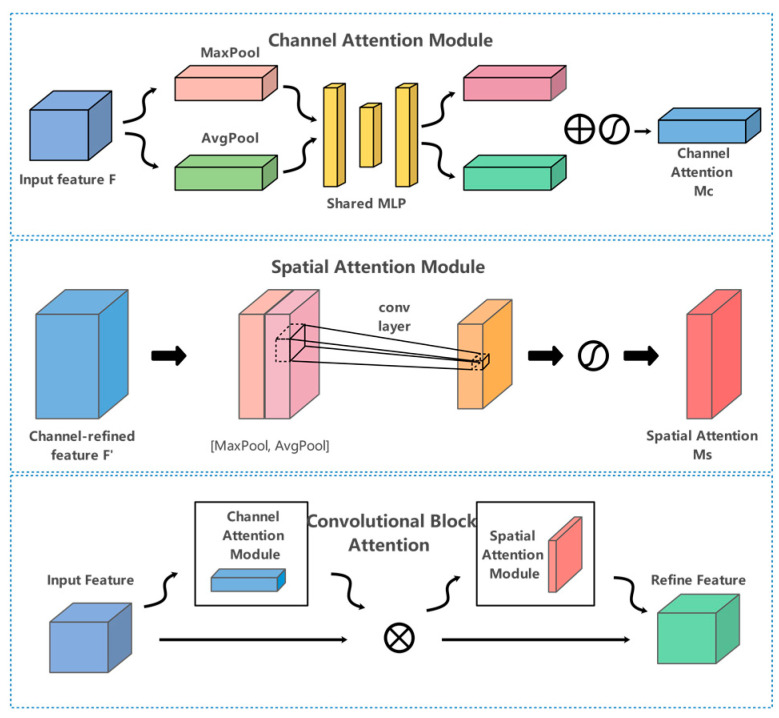
Convolutional Block Attention module.

To address the challenging problem of the fuzzy boundary of drivable areas, we used the CBAM module to redistribute feature weights after the first up-sampling operation as shown in Figure 4. This was achieved by inferring the attention map along two independent dimensions (channel and space) and then multiplying it with the input feature map to perform adaptive feature optimization. This step was necessary to enable the segmentation decoding module to focus more on the fuzzy boundary of drivable areas.

### 3.4. Loss Function

Our framework performed object detection and drivable area segmentation simultaneously, and our loss function included the object detection loss and the drivable area segmentation loss.

The object detection loss $L_{\det}$ contains classification loss $L_{\mathrm{cls}}$, object loss $L_{\mathrm{obj}}$ and bounding box loss $L_{\mathrm{bbx}}$. $L_{\mathrm{cls}}$ and $L_{\mathrm{obj}}$ are both binary cross-entropy loss, which is used to minimize the error of classification and of the confidence of one prediction separately. We used SIoU loss [38] as $L_{\mathrm{bbx}}$, which takes the distance, overlap rate, the similarity of shape and orientation between the predicted box and ground truth into account, while most other loss functions do not consider orientation.

The definition of SIoU loss is:

(7) $${\mathrm{Loss}}_{\mathrm{SIoU}}=1-\mathrm{IoU}+\frac{\Delta +\Omega }{2}$$

The angle cost Λ is:

(8) $${\Lambda =1-2\sin}^{2}\left(\arcsin(x)-\frac{\pi }{4}\right)$$

The distance cost Δ is:

(9) $$\Delta =\underset{t=x,y}{\sum }\left(1-e^{{-\gamma \rho }_{t}}\right)$$

where γ=2−Λ.

The shape cost Ω is

(10) $$\Omega =\underset{t=w,h}{\sum }{\left(1-e^{-\omega_{t}}\right)}^{\theta }$$

The IoU cost is:

(11) $$\mathrm{IoU}=\frac{/B\cap B^{\mathrm{GT}}/}{/B\cup B^{\mathrm{GT}}/}$$

As Equation (12), the $L_{\det}$ is the weight sum of three loss above.

(12) $$L_{\det}=\alpha_{1}L_{\mathrm{cls}}+\alpha_{2}L_{\mathrm{obj}}+\alpha_{3}L_{\mathrm{bbx}}$$

The drivable area segmentation loss $L_{\mathrm{seg}}$ is binary cross-entropy loss, which is used to penalize the classification of each pixel in th Modified e segmentation map.

In conclusion, the final loss function of our network is given by.

(13) $${L=\alpha L}_{\det}{+\beta L}_{\mathrm{seg}}$$

where α and β are the weight of detection loss and segmentation loss.

We can tune $\alpha_{1}$, $\alpha_{2}$, $\alpha_{3}$ to balance the three parts of detection task and tune α, β to balance the detection part and segmentation part.

## 4. Experiment

In this section, we demonstrate the details of our experiment and that all our experiments in this paper were conducted based on Intel Xeon E5-2698 v4 and one 32G NVIDIA TESLA V100. The construction of the experimental model was based on PyTorch.

### 4.1. Dataset

**Figure 5:**
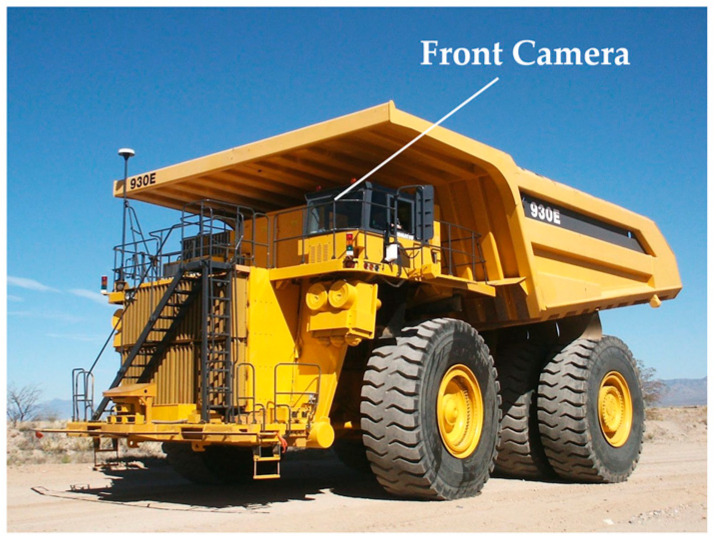
Data collection platform and the position of front camera.

Currently, there are limited datasets for object detection and segmentation specifically tailored for mining scenes. To validate our algorithm, we constructed our own mining perception dataset called Minescape. According to the actual mining operation scenario, we used a XDE240 mining truck as the data collection platform and its FLIR Point Grey Flea front camera as the image sensor with 10Hz capture frequency as shown in Figure 5. The Minescape consisted of 4547 training images, 1299 verification images and 650 test images with various mine scenes, 60+ driving hours of three days and varying luminance conditions from Shenyan Coal and Xiwan Opencast Coal Mines. To ensure the accuracy of annotations, we used labelme to manually annotate the images. We evaluated our network on the Minescape verification set.

The Minescape had 9 types of label objects, including 4 types of movable objects (mining truck, car, person, engineering vehicle) and 5 types of static objects (stone, other obstacle, sign, background, drivable area), as shown in Table 1. These categories cover most of the work scenes of mining trucks, and their scale and number distribution is very uneven, which is one of the reasons why the general detection framework is not robust in the mining environment.

**Figure 6:**
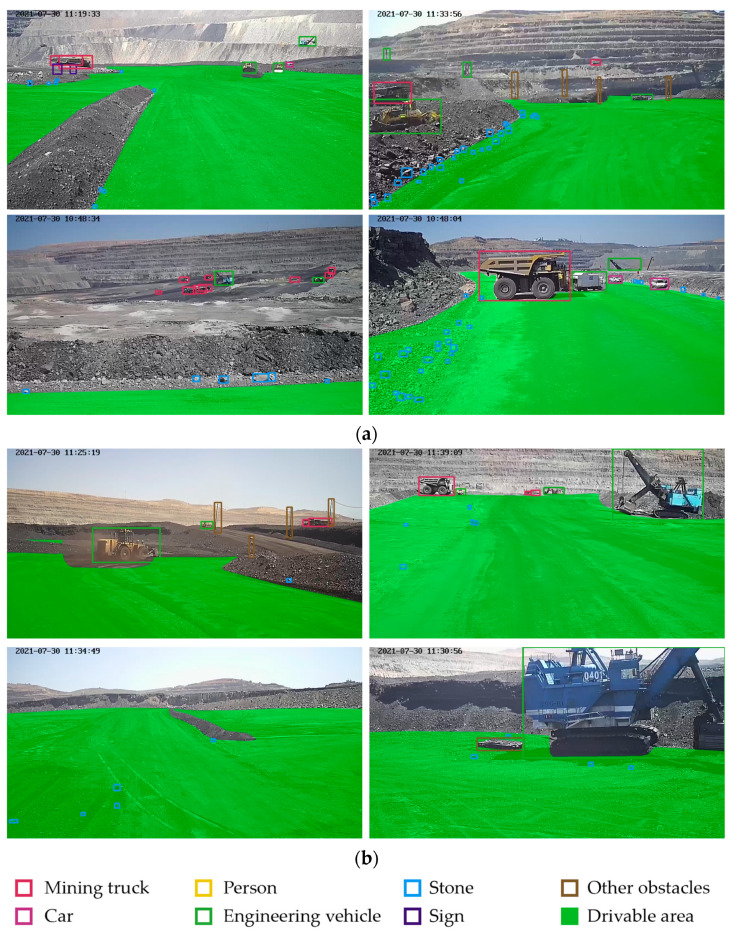
Some representative pictures in Minescape. (**a**) The distribution of small objects in the mine. They are either aggregated or dispersed. They are arranged irregularly and have strong camouflage. (**b**) The characteristics of the drivable area. The boundary shape of the drivable area is irregular and sometimes discontinuous. Its color and texture features are also unstable.

It is worth mentioning that the advantage of the Minescape is that it reveals some common problems in open-pit mine, such as clustered small objects and the unobvious boundary of drivable areas, as shown in Figure 6. The Minescape provided us with data in various typical scenarios to design a framework to solve such problems.

### 4.2. Metrics

The algorithm proposed by us included two tasks: detection and segmentation. A general evaluation index can judge the performance of the model, which is also a visual manifestation of the comparison and evaluation with existing models. At present, the evaluation indexes of target detection algorithm widely accepted by many scholars include Loss Function, Intersection over Union (IoU) and mean Average Accuracy (mAP). The evaluation indicators of segmentation tasks mainly include Mean Intersection over Union (MIoU) and Accuracy (Acc).

#### 4.2.1. Evaluation Metrics of Target Detection

The intersection over union (IoU) is an important reference index in the field of target detection, which represents the overlap rate between candidate boxes and tag boxes. When IoU value is 1, it indicates that candidate box and marker box are completely coincident, and the formula is as follows:

(14) $$\mathrm{IoU}=\frac{A\cap B}{A\cup B}$$

Precision refers to the proportion of real positive cases among the samples predicted as positive cases, expressed as *P*. Formula is shown as follows:

(15) $$p=\frac{\mathrm{TP}}{\mathrm{TP}+\mathrm{FP}}$$

Recall rate refers to the proportion of correctly predicted samples in all actually positive samples. The formula is as follows:

(16) $$\mathrm{recal}=\frac{\mathrm{TP}}{\mathrm{TP}+\mathrm{FN}}$$

Average Precision (AP) means the average precision rate under different recall rates, expressed as the formula:

(17) $$\mathrm{AP}=\overset{n}{\underset{i=1}{\sum }}P(i)∆r(i)=\int_{0}^{1}p(r)\mathrm{dr}$$

The definition of Mean Average Precision (mAP) is to calculate the mean of AP, which is the average precision of all categories. The formula is as follows:

(18) $$\mathrm{mAP}=\frac{\sum_{n=1}^{N}\mathrm{AP}(n)}{N}$$

in the formula, represents the category, and represents the total number of categories.

#### 4.2.2. Evaluation Metrics for Task Segmentation

IoU represents the ratio of the intersection and union of the two sets of the real area and the prediction area, and MIoU represents to calculate the IoU of each category, then sum and average. The calculation formula of MIoU is:

(19) $$\mathrm{MIoU}=\frac{1}{k}\overset{\kappa }{\underset{i=1}{\sum }}\frac{V_{\mathrm{Ti}}\cap V_{\mathrm{Pi}}}{V_{\mathrm{Ti}}\cup V_{\mathrm{Pi}}}$$

Pixel Accuracy represents the percentage of correctly classified pixels in the image. It’s given by:

(20) $$\mathrm{Accuracy}=\frac{\mathrm{TP}+\mathrm{TN}}{\mathrm{TP}+\mathrm{TN}+\mathrm{FT}+\mathrm{FN}}$$

### 4.3. Implementation Detail

We used SGD with momentum as the optimizer, with an initial learning rate of 0.01. To better train our model, we performed 3 epochs of warm up with an initial learning rate of 0.1. We trained 80 epochs on our dataset with the batch size of 16 and the input image size of (640, 640, 3). In the final loss function settings, the parameters $\alpha_{1}$, $\alpha_{2}$, $\alpha_{3}$, α and β as mentioned before were 0.5, 2.0, 0.1, 1.0 and 0.8, respectively.

### 4.4. Experiment Results

We developed an end-to-end multi-task network. However, it was difficult to find a single model that can fulfill all multi-task requirements. Currently, a model that performs well in object detection may not perform well in segmentation. Therefore, we compared the performance of our model with the state-of-the-art (SOTA) algorithms both in object detection and semantic segmentation. The results are shown in Table 2.

To evaluate small object detection, we compared our model with SOTA object detection algorithms such as YOLOv7tiny, YOLOv5n and Faster-RCNN. Our dataset consisted of small objects such as stones, signs and other obstacles, and we reported mAP results for the above algorithms, which ranged from 32.2% to 73.3%. Our model achieved significantly higher results of 67.6%/87.5%/86.5%, with the highest improvement of 27.0%/18.5%/13.2% over the SOTA algorithms. Interestingly, our model used the same backbone as YOLOv7tiny, but with the addition of Multi-head Feature Fused Detector and CAM-SimAM, we were able to extract better small object features.

We also conducted a comparison experiment of segmentation networks using the same experimental setup. We compared three networks: Bisnet, U-Net and HarDNet, which are the baseline algorithms for classic segmentation networks and drivable area segmentation algorithms. We used two evaluation indicators, MIoU and Acc, and our algorithm achieved higher results of 0.6850/0.8150, which were 1% higher than each other. The results are shown in Table 3.

**Figure 7:**
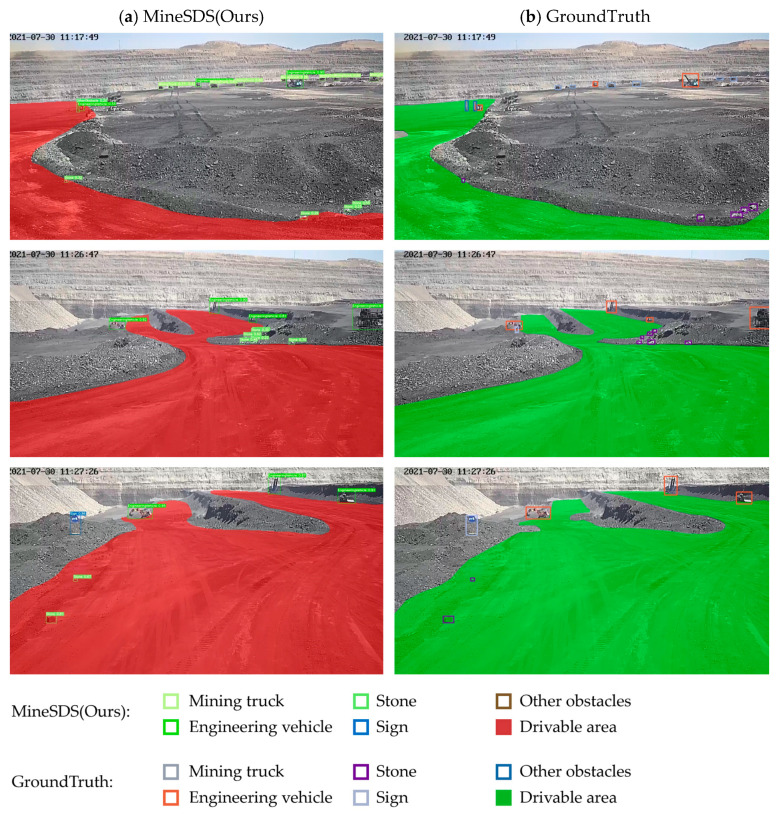
Results on Minescape.

We visualized the prediction results of our model on Minescape, and some of the results are shown in Figure 7. It can be seen that our model can detect the objects very well, and it also showed comparatively well for the small objects, but there were still, sometimes, missing detections when comparing with the ground truth. In terms of segmentation, comparing with the ground truth, it can basically segment the boundary correctly, and the segmentation effect became worse at the farther distance.

### 4.5. Ablation Study

The purpose of conducting ablation experiments is to explore the contribution of various components within our framework. We conducted corresponding experiments to explore the role of attention components and loss functions. Additionally, we trained our model to perform both object detection and drivable area segmentation tasks separately to assess the effectiveness of our multi-task training approach.

Our experiments and their corresponding results are presented in Table 4. The results demonstrate that the attention module significantly improved the model’s overall mAP by 2.9%, with significant improvements seen in small objects such as stones, other obstacles and signs. On the other hand, there was no significant difference observed in the improvement of other categories with normal scale. Our multi-task training framework resulted in a 1.5% increase in overall mAP compared to training solely for the detection task. However, the difference between the result of training for the segmentation task individually and that of multi-task training was not apparent. Experimentally, we found that the SIoU loss function was more favorable to our model’s training than CIoU, with the model trained with SIoU yielding a 2.2% higher mAP than the model trained with CIoU.

## 5. Conclusions

In this paper, we proposed a perceptual framework for mining scenarios that focused on improving small object detection and drivable area segmentation. To achieve this, we employed a small object attention module that enhanced the network’s accuracy for detecting numerous small objects. Additionally, we used CBAM to improve the feature mapping capabilities of convolutional operations, allowing our network to focus on the fuzzy boundaries of drivable areas. We also trained both tasks simultaneously using a weighted loss function. Our extensive experiments demonstrated that our framework performed comparably well. However, our work had some limitations. Firstly, the data we collected did not include bad weather conditions, which could impact the robustness of the algorithm in real-world applications. To address this, we will need to re-collect a substantial amount of data. Moreover, we did not operate our algorithm and other algorithms on a classical benchmark dataset. This may be biased. We will compare our algorithm with state-of-the-art methods on a more representative benchmark dataset.

## Figures and Tables

**Table 1:** Detail of classes in Minescape.

Group	Class	Quantity	Description
movable objects	mining truck	4845	mining dump trucks
	car	601	cars and pickups
	person	2965	workers
	engineering vehicle	5638	excavator, etc.
static objects	stone	136,578	ore and rock
	sign	1205	traffic signs
	other obstacles	2575	other common obstacles
	drivable area	9376	safe drivable ground
	background	3035	undrivable area

**Table 2:** Evaluation results of different detection algorithms on Minescape.

Model	Backbone	mAP0.5 (%)							
		All	Mining Truck	Other Obstacle	Car	Person	Engineering Vehicle	Stone	Sign
YOLOv7 tiny	ELAN	78.5	90.8	70.4	89.4	95.6	96.9	40.6	66.0
YOLOv5n	CSPNet	77.3	88.4	69.5	89.1	89.4	95.3	40.4	69.0
Faster-RCNN	ResNet-50	76.8	87.8	73.3	92.5	89.1	95.5	32.2	67.0
MineSDS-det (ours)	ELAN	87.8	95.7	86.5	94.9	94.9	98.3	67.6	87.5

**Table 3:** Evaluation results of different segmentation algorithms on Minescape.

Network	MloU	Accuracy
Bisnet	0.6450	0.7842
U-Net	0.6571	0.7931
HarDNet	0.6775	0.8079
MineSDS-seg (ours)	0.6850	0.8150

**Table 4:** Ablation studies of MineSDS.

Method	mAP0.5 (%)								mloU (%)	Accuracy
	All	Mining Truck	Other Obstacle	Car	Person	Engineering Vehicle	Stone	Sign		
X (base)	84.9	95.7	80.9	95.1	94.3	97.7	52.6	77.6	68.2	81.3
Y (X + SSWAM)	87.8	95.7	86.5	94.9	93.9	98.3	57.6	87.5	68.5	81.5
Y-Det (only)	86.3	95.2	83.2	93.9	93.8	97.8	55.8	84.4	-	-
Y-Seg (only)	—	—	—	—	—	—	—	—	68.4	81.5
CIoU Loss	77.6	90.4	73.4	87.6	93.1	96.3	44.8	57.7	68.3	81.4
SIoU Loss	81.7	92.6	79.5	90.7	94.3	97.5	45.8	71.7	68.4	81.4

## Data Availability

The data presented in this study are available on request from the corresponding author. The data are not publicly available due to third party (data source) restrictions.
